# The Sooner, the Better? The Importance of Optimal Timing of Cholecystectomy in Acute Cholecystitis: Data from the National Swedish Registry for Gallstone Surgery, GallRiks

**DOI:** 10.1007/s11605-016-3223-y

**Published:** 2016-09-20

**Authors:** My Blohm, Johanna Österberg, Gabriel Sandblom, Lars Lundell, Mats Hedberg, Lars Enochsson

**Affiliations:** 1 Division of Surgery, Department of Clinical Science, Intervention and Technology, Karolinska Institutet, Stockholm, Sweden; 2 Department of Surgery, Mora Hospital, 792 85 Mora, Sweden; 3 Center for Digestive Diseases, Karolinska University Hospital, 141 86 Stockholm, Sweden

**Keywords:** Acute cholecystitis, Laparoscopic cholecystectomy, Open cholecystectomy, Admission day, Adverse events, Bile duct injury

## Abstract

Up-front cholecystectomy is the recommended therapy for acute cholecystitis (AC). However, the scientific basis for the definition of the optimal timing for surgery is scarce. The aim of this study was to analyze how the timing of surgery, after the admission to hospital for AC, affects the intra- and postoperative outcomes. Within the national Swedish Registry for Gallstone Surgery and Endoscopic Retrograde Cholangiopancreatography (GallRiks), all patients undergoing cholecystectomy for acute cholecystitis between January 2006 and December 2014 were identified. Data regarding patient characteristics, intra- and postoperative adverse events (AEs), bile duct injuries, and 30- and 90-day mortality risk were captured, and the correlation between the surgical timing and these parameters was analyzed. In total, data on 87,108 cholecystectomies were analyzed of which 15,760 (18.1 %) were performed due to AC. Bile duct injury, 30- and 90-day mortality risk, and intra- and postoperative AEs were significantly higher if the time from admission to surgery exceeded 4 days. The time course between surgery and complication risks seemed to be optimal if surgery was done within 2 days after hospital admission. Although AC patients operated on the day of hospital admission had a slightly increased AE rate as well as 30- and 90-day mortality rates than those operated during the interval of 1–2 days after admission, the bile duct injury and conversion rates were, in fact, significantly lower. The optimal timing of cholecystectomy for patients with AC seems to be within 2 days after admission. However, the somewhat higher frequency of AE on admission day may emphasize the importance of optimizing the patient before surgery as well as ensuring that adequate surgical resources are available.

## Introduction

Cholecystectomy has since long been the therapy of choice for elective treatment of patients with symptomatic gallstone disease. Acute cholecystitis (AC) is a well-known complication of gallbladder stone disease. Over the years, it has been claimed that AC can be primarily treated conservatively and then followed by delayed elective cholecystectomy. In recent decades, evidence has been gathered to show that early cholecystectomy for AC during the acute hospital stay is safe and cost-effective.^1^–^11^ Lately, data have emerged from centers with great experience in laparoscopic surgery to show that early laparoscopic cholecystectomy is an attractive, feasible, and safe therapeutic strategy.^12^–^14^ Recent meta-analyses concluded that early laparoscopic cholecystectomy appears to be safe and reduces total hospital stay.^15^–^17^ Similar conclusions were also reached in a current Cochrane systemic review.^18^ A recent multicenter (*n* = 35 centers) randomized trial (*n* = 618 patients), studying early versus delayed cholecystectomy, finally settled the issue showing that laparoscopic cholecystectomy, when performed within 24 h of hospital admission, was significantly better as compared to conservative treatment regarding both morbidity and costs.^13^ Another controversial topic is the optimal timing of surgery in AC, where one of the issues is whether it should be operated early (i.e., within the first 48 to 96 h) or delayed (within the same hospital stay), depending on the actual practical and logistic circumstances. Studies, incorporating a limited number of patients, have indicated that the best time window to operate AC might be within the first 48 to 96 h.^5^,^8^ A French nationwide registry study has recently shown that operation within 3 days after admission is recommended in patients with acute cholecystitis.^19^ To address a similar question within an RCT, it would require a large number of patients to create sufficient power for testing the hypotheses, which basically preclude the completion of trials within reasonable time limits. National quality registry studies can act as a complement to RCTs. Registries enable data collection on large-scale patient cohorts, permitting analyses with good statistical power. Corresponding registry studies have been able to address clinical questions that, due to statistical, time, and financial constraints, would never have been studied in RCTs, for example the value of intraoperative cholangiography in preventing bile duct injury in gallstone surgery.^20^–^21^ The aim of this study was therefore to compare the outcomes for patients operated on for AC in the entire country, during a 9-year period, with particular focus on the time elapsed from admission to hospital until the cholecystectomy was carried out, and the impact of these time periods on the intra- and postoperative adverse events.

## Methods

### Swedish Registry of Gallstone Surgery and Endoscopic Retrograde Cholangiopancreatography (GallRiks)

The Swedish Registry of Gallstone Surgery and Endoscopic Retrograde Cholangiopancreatography (ERCP) was founded on May 2005 by the Swedish National Board of Health and Welfare, the Swedish Surgical Association, and the Swedish Society of Laparoscopic Surgery. It is financially supported by the Swedish national health authorities. On an annual basis, approximately 20,000 procedures (12,000 cholecystectomies and 8000 ERCPs) are registered. The aim of the registry is to obtain continuously updated information regarding indications, outcomes, and patient satisfaction in order to ensure high-quality care of patients needing cholecystectomy, regardless of where in Sweden they are managed.^22^ The operating surgeon registers the procedure online, preferably during the operation or immediately after. A 30-day follow-up is administered by the local coordinator at each participating hospital. The follow-up is also registered online. The validity of data is monitored by independent reviewers, who visit the participating hospitals at least once every third year and compare the online registrations with the corresponding patient records. The validation of GallRiks has demonstrated high correctness and completeness of data and no failure to report serious adverse events.^23^ The registry attained an overall national coverage of approximately 90 % during the actual enrolment period.

### Study Design

**Fig. 1:**
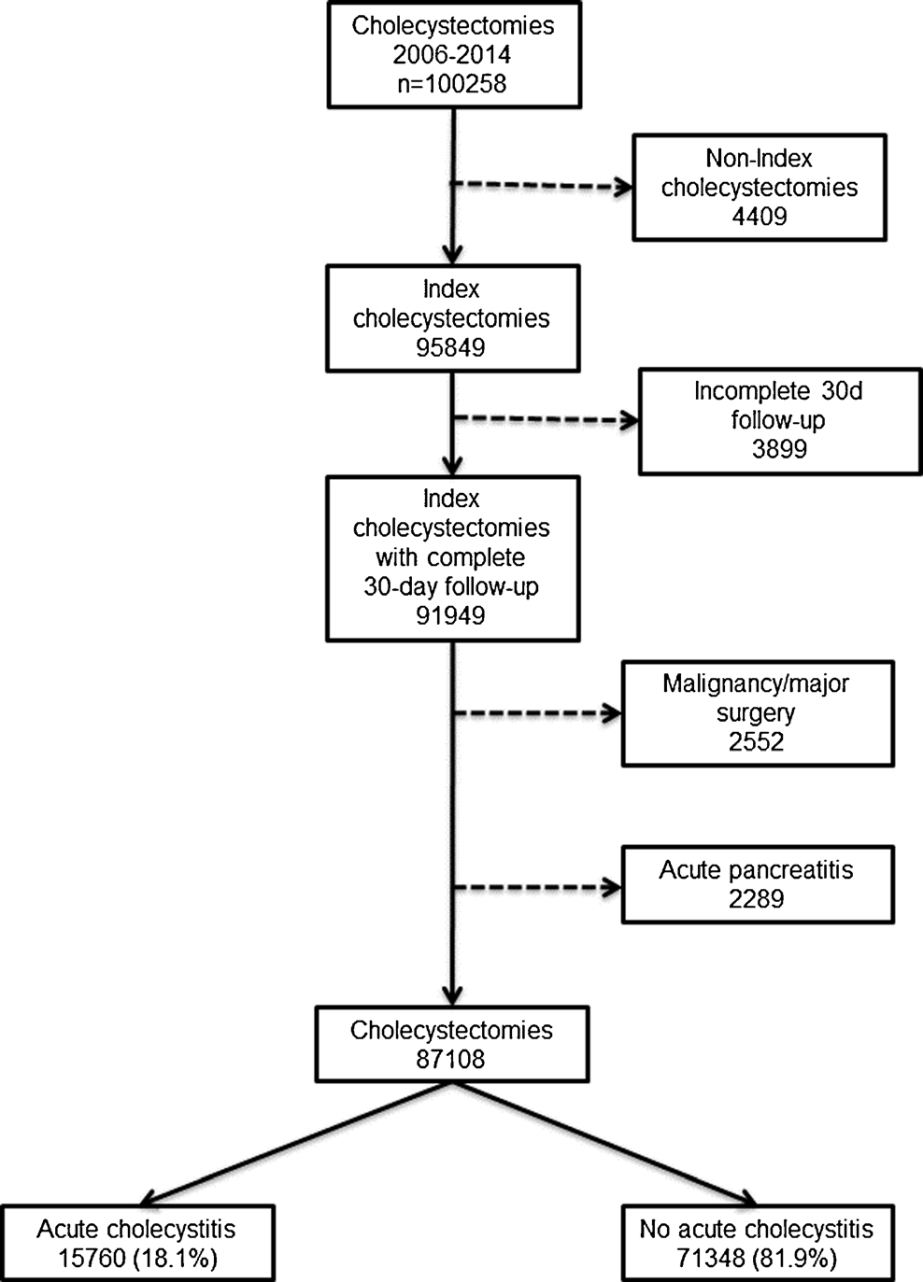
The procedures included in the analysis

We conducted a nationwide, population-based, nested case-control study within the cohort of cholecystectomies entered into GallRiks between 1 January 2006 and 31 December 2014. Index cholecystectomies with a complete 30-day follow-up were identified. From this cohort, procedures performed with the indications of malignancy, concomitant major surgery in conjunction with cholecystectomy, as well as acute pancreatitis were excluded, leaving 87,108 cholecystectomies for analysis (Fig. 1).

### Definitions

For the purpose of this paper and in accordance with the descriptions in the GallRiks database, the following definitions were applied.

- *Intraoperative adverse events* are defined as bleeding, bile duct injury, gut perforation, or any other reason for the cholecystectomy to be terminated prematurely.
- *Intraoperative bleeding demanding intervention* is defined as any bleeding requiring blood transfusion or conversion to open surgery. Bleeding that is controlled completely with diathermy, clips, or sutures is not considered to be intraoperative bleeding demanding an intervention. Data are not filed on the volume of blood loss and number of transfused blood units.
- *Bile duct injury* (*BDI*) is defined as intraoperatively registered (a) BDI ≤1/3 of the diameter, (b) BDI >1/3 of the diameter, (c) resection of a substantial part of the bile duct wall, or (d) complete transection of the bile duct.
- *Postoperative adverse events* are defined as any complication during the 30-day follow-up period that required some form of medical or surgical intervention.

### Statistics

Statistical analysis was performed using JMP 12.1.0 (64-bit) (SAS Institute, Inc., Cary, NC, USA). Comparisons of patient- and procedure-related characteristics were presented in contingency tables, with pairwise differences analyzed with Pearson’s chi-square test and presented as *P* values. The association between acute cholecystitis and the time of operation after admission as well as the risk of adverse events and mortality were analyzed using multivariable logistic regression modeling. Variables that were statistically significant in univariate analysis were included in the multivariate model as described by Hosmer et al.^24^

In the multivariate analysis, the outcome was adjusted for age (dichotomized into more or less than 50 years), gender, comorbidity (dichotomized into ASA 1–2 and ASA ≥3), acute or scheduled surgery, indication for and type of surgery, as well as if the patients had a history of previous acute cholecystitis.

The models were tested for multicollinearity and effect modification and finally assessed for goodness of fit. The effects of analyzed variables were presented as odds ratios (ORs) for adverse events with 95 % confidence intervals.

### Ethical considerations

The regional research ethics committee at Karolinska Institutet, Stockholm, Sweden, approved the study.

## Results

**Fig. 2:**
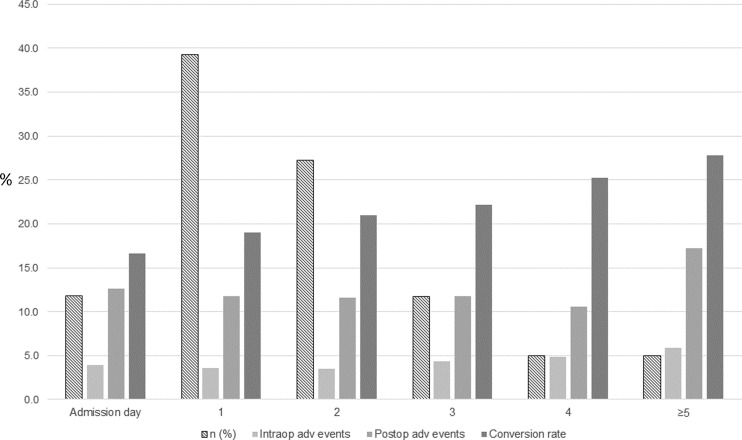
Acute cholecystitis (AC). Adverse events in relation to the time of surgery after admission

Between 1 January 2006 and 31 December 2014, 100,258 cholecystectomies were registered in GallRiks of which 87,108 met the inclusion criteria (Fig. 1). The demographics of the study population, the surgical technique for the cholecystectomy, antibiotic use, and length of hospital stay for patients with or without AC are given in Table 1. For all outcome measures given, except for the length of postoperative hospital stay after open cholecystectomy, the two groups (AC versus no AC) differed significantly. In the AC group, there was a significantly increased risk of intra- and postoperative adverse events (Table 2). Neither the 30- nor the 90-day mortality risk, however, differed significantly in the multivariate analysis (Table 2). In the subgroup of patients operated on with cholecystectomy (laparoscopic or open) for AC, the majority had an operation during the first day after admission (39.2 %) followed by those who had an operation on the second day (27.3 %). Cholecystectomy on the admission day was performed in 11.9 % of the AC patients (Fig. 2). The conversion rate in the latter group increased in a stepwise manner from 16.6 % on admission day to 27.8 % on >4 days after admission (Fig. 2). The multivariate analysis of the AC patients, with regard to the risk of intraoperative adverse events, revealed a significantly lower risk for adverse event (AE) when the operation was done on the first or second day after admission (Table 3). The risk of intraoperatively detected bile duct injuries was, however, lowest when the cholecystectomy was done on the day of admission with a thereafter day-by-day stepwise increased risk (Table 3). The risk of postoperative AE was significantly lower in patients operated within the first 4 days after admission as compared to those operated 5 days or more after admission (Table 3). The 30-day mortality risk was significantly lower when operated during the first day after admission, and even the 90-day mortality risk was significantly reduced if the operation was completed within 3 days of admission as compared to those operated 5 days or more thereafter (Table 3). Intraoperative blood loss was not affected by the time elapsed from admission.

**Table 1:** Demographics of acute cholecystitis (AC) and no AC

	AC (*n* = 15,760)		No AC (*n* = 71,348)		*P*
	*n*	%	*n*	%	
Gender					
Females	8376	53.15	50,526	70.82	*<0.0001*
Males	7384	46.85	20,822	29.18	
Age (years)^a^					
>50	9731	61.97	33,073	46.42	*<0.0001*
≤50	5971	38.03	38,169	53.58	
ASA					
1–2	13,792	87.51	66,965	93.86	*<0.0001*
≥3	1968	12.49	4383	6.14	
Op technique					
LC (Op started with laparoscopic technique)	12,522	79.45	68,803	96.43	*<0.0001*
Converted^b^	2520	20.12	3622	5.26	*<0.0001*
Antibiotics					
Prophylactic	4531	28.75	11,859	16.62	*<0.0001*
Treatment	7963	50.53	3002	4.21	
No antibiotics	3266	20.72	56,487	79.17	
Hospital stay (days)^c^	Mean	SEM	Mean	SEM	
Length of postop stay	3.52	0.04	1.59	0.01	*<0.0001*
LC postop stay	2.98	0.04	1.43	0.01	*<0.0001*
OC postop stay	5.60	0.11	5.69	0.15	0.5988

^a^Missing 164

^b^Excluded 5783 OCs

^c^Missing 85

**Table 2:** Risk of intra- and postoperative adverse events (AEs) in acute cholecystitis (AC) and no AC, respectively

	Number		Percent	Univariate		Multivariate^a^	
	AE	No AE		OR (95 % CI)	*P*	OR (95 % CI)	*P*
Intraop AE							
No AC	2028	69,320	2.84	1.0 (reference)		1.0 (reference)	
AC	618	15,142	3.92	1.40 (1.27–1.53)	*<0.0001*	1.23 (1.12–1.35)	*<0.0001*
Intraop bleeding							
No AC	496	70,852	0.70	1.0 (reference)		1.0 (reference)	
AC	234	15,526	1.48	2.15 (1.84–2.51)	*<0.0001*	1.54 (1.30–1.82)	*<0.0001*
Intraop bile duct injury							
No AC	201	71,147	0.28	1.0 (reference)		1.0 (reference)	
AC	67	15,693	0.43	1.51 (1.14–1.98)	*0.0048*	1.44 (1.08–1.90)	*0.0137*
Postop AE							
No AC	5250	66,098	7.36	1.0 (reference)		1.0 (reference)	
AC	1896	13,864	12.03	1.72 (1.63–1.82)	*<0.0001*	1.38 (1.26–1.51)	*<0.0001*
Mortality (≤30 days)							
No AC	47	71,301	0.07	1.0 (reference)		1.0 (reference)	
AC	73	15,687	0.46	7.06 (4.91–10.25)	*<0.0001*	1.25 (0.67–2.22)	0.4720
Mortality (≤90 days)							
No AC	100	71,248	0.14	1.0 (reference)		1.0 (reference)	
AC	129	15,631	0.82	5.88 (4.53–7.65)	*<0.0001*	1.04 (0.72–1.49)	0.8137

^a^Adjusted for sex, age, ASA, acute/scheduled surgery, indication, type of surgery, and previous acute cholecystitis

**Table 3:** Risk of adverse events (AEs) in acute cholecystitis in relation to the timing of surgery after admission to hospital

Time between admission and treatment (days)	Number (*n* = 15,189^a^)			Univariate		Multivariate^a^	
	Yes	No	%	OR (95 % CI)	*P*	OR (95 % CI)	*P*
Intraoperative AE							
≥5	44	709	5.84	1.0 (reference)		1.0 (reference)	
4	37	718	4.90	0.83 (0.53–1.30)	0.4166	0.88 (0.56–1.38)	0.5819
3	78	1703	4.38	0.74 (0.51–1.09)	0.1221	0.81 (0.55–1.19)	0.2811
2	146	3997	3.52	0.59 (0.42–0.84)	***0.0041***	0.66 (0.47–0.95)	*0.0261*
1	212	5745	3.56	0.59 (0.43–0.84)	***0.0038***	0.67 (0.49–0.96)	***0.0285***
Admission day	71	1729	3.94	0.66 (0.45–0.98)	***0.0393***	0.74 (0.50–1.10)	0.1377
Intraoperative bleeding							
≥5	15	738	1.99	1.0 (reference)		1.0 (reference)	
4	15	740	1.99	1.00 (0.48–2.07)	0.9941	1.14 (0.55–2.38)	0.7254
3	29	1752	1.63	0.81 (0.44–1.57)	0.5271	0.97 (0.52–1.88)	0.9187
2	61	4082	1.47	0.74 (0.43–1.35)	0.3052	0.94 (0.54–1.73)	0.8347
1	80	5877	1.34	0.67 (0.40–1.21)	0.1771	0.86 (0.50–1.57)	0.6054
Admission day	28	1772	1.56	0.78 (0.42–1.50)	0.4417	0.97 (0.52–1.88)	0.9255
Bile duct injury intraoperatively							
≥5	7	746	0.93	1.0 (reference)		1.0 (reference)	
4	4	751	0.53	0.57 (0.15–1.89)	0.3586	0.59 (0.15–1.97)	0.3950
3	12	1769	0.67	0.72 (0.29–1.95)	0.5037	0.78 (0.31–2.11)	0.6106
2	16	4127	0.39	0.41 (0.18–1.08)	*0.0691*	0.45 (0.19–1.18)	0.1007
1	22	5935	0.37	0.40 (0.18–1.00)	*0.0505*	0.43 (0.19–1.11)	*0.0780*
Admission day	3	1797	0.17	0.18 (0.04–0.64)	***0.0081***	0.19 (0.04–0.68)	***0.0107***
Postoperative AE							
≥5	130	623	17.26	1.0 (reference)		1.0 (reference)	
4	80	675	10.60	0.57 (0.42–0.76)	***0.0002***	0.61 (0.45–0.82)	***0.0012***
3	209	1572	11.73	0.64 (0.50–0.81)	***0.0002***	0.71 (0.56–0.90)	***0.0055***
2	480	3663	11.59	0.63 (0.51–0.78)	***<0.0001***	0.72 (0.58–0.90)	***0.0041***
1	700	5257	11.75	0.64 (0.52–0.79)	***<0.0001***	0.73 (0.60–0.91)	***0.0044***
Admission day	227	1573	12.61	0.69 (0.55–0.88)	***0.0024***	0.78 (0.62–1.00)	***0.0468***
Mortality (≤30 days)							
≥5	10	743	1.33	1.0 (reference)		1.0 (reference)	
4	10	745	1.32	1.00 (0.41–2.44)	0.9952	1.36 (0.54–3.43)	0.5030
3	8	1773	0.45	0.34 (0.13–0.85)	***0.0223***	0.49 (0.18–1.28)	0.1465
2	16	4127	0.39	0.29 (0.13–0.66)	***0.0042***	0.52 (0.23–1.21)	0.1245
1	18	5939	0.30	0.23 (0.11–0.51)	***0.0007***	0.40 (0.18–0.93)	***0.0344***
Admission day	10	1790	0.56	0.42 (0.17–1.02)	***0.0539***	0.62 (0.25–1.56)	0.3096
Mortality (≤90 days)							
≥5	22	731	2.92	1.0 (reference)		1.0 (reference)	
4	13	742	1.72	0.58 (0.28–1.15)	0.1198	0.77 (0.37–1.57)	0.4794
3	15	1766	0.84	0.28 (0.14–0.54)	***0.0002***	0.40 (0.20–0.79)	***0.0085***
2	30	4113	0.72	0.24 (0.14–0.43)	***<0.0001***	0.42 (0.24–0.76)	***0.0048***
1	29	5928	0.49	0.16 (0.09–0.29)	***<0.0001***	0.28 (0.16–0.51)	***<0.0001***
Admission day	17	1783	0.94	0.32 (0.16–0.60)	***0.0004***	0.47 (0.24–0.91)	***0.0253***

In 571 of the 15,760, LOS was not correctly stated. Values in bold-italic are statistically significant (*P*<0.05). Values in italic are not statistically significant but indicate a trend

^a^Adjusted for sex, age, ASA, type of surgery, and previous acute cholecystitis

## Discussion

This study represents one of the largest patient cohorts addressing the correlation between the time elapsed from hospital admission for AC and definitive surgery and the risk for intra- and postoperative AE. We found that the optimal time slot for surgery was within the first 2 days after hospital admission. Cholecystectomy on admission day was performed in close to 12 % of the AC patients, and the risk of intraoperatively detected bile duct injuries was lowest when the cholecystectomy was done on the day of admission with a thereafter day-by-day stepwise increase. These findings support the findings in the literature of the beneficial effect of early surgery on AC.^5^,^8^,^19^ This information is clinically relevant since the majority of minor bile duct lesions are detected intraoperatively and repair is usually attempted at the index procedure with satisfactory results,^25^ an observation which is not entirely consistent with the international experiences.^20^,^21^ However, in the multivariate analysis, we observed a significantly lower risk for AE when the operation was done on the first or second day after admission. These differences in morbidity were also translated into 30-day mortality risk, which were significantly lower when the cholecystectomy was completed during the first day after admission. Even the 90-day mortality risk was significantly reduced for those operated within 3 days of admission. Although operation on admission day in most of our assessments has a significantly better outcome than in those being operated 5 days or more after admission, the frequency of adverse events was somewhat higher compared to an operation performed 1 or 2 days thereafter. The reason for these findings may be found in the fact that some patients arrive at the emergency unit in a bad general condition where time is required for resuscitation and stabilization of the patient, before embarking on definitive surgery. Furthermore, if the patient with acute cholecystitis arrives at the hospital during non-office hours and is immediately scheduled for cholecystectomy, the issue of not being able to offer the highest level of laparoscopic surgical expertise may have a bearing on the outcome. Nevertheless, these issues did not translate into an enhanced risk for BDI.

In a similar analysis of 4113 prospective cholecystectomies performed on patients with acute cholecystitis as reported from the Swiss Association of Laparoscopic and Thoracoscopic Surgery (SALTS) database, the authors concluded that immediate surgery (surgery on the day of admission or at the latest within 24 h of admission) significantly reduced the risk of conversion from laparoscopic to open surgery, which can be used as a proxy variable for the safety and accuracy of the respective operations.^12^ This is to some extent in line with our study, where the conversion rate, when operation occurred on admission day, was 16.6 % and then increased in a linear fashion to 27.8 % in patients operated on 4 days later. However, whereas the technical conditions for surgery may be optimal on admission day, due to less extent of the inflammation, the findings in our study of a better outcome when patients are operated on the first or second day after admission may indicate that other factors, like the general condition of the patient, mandate attention in order to optimize the outcome.

Studies on the management of patients with AC in routine surgical practice have revealed that basically, no major changes have occurred over recent years.^14^,^26^,^27^ This illustrates the problems and challenges in the implementation of evidence-based guidelines for the treatment of AC. To some extent, this may be explained by a larger proportion of elderly patients with high comorbidity; however, one cannot rule out a widespread conservative attitude with resistance to new evidence-based approaches.^26^ The slow adaptation to new guidelines could have several other explanations such as local logistic problems and lack of resources for acute surgery. There may also be a proportion of patients with a long duration of symptoms before seeking medical attention, resulting in reluctance to an emergency operation.

The strengths of the current analyses are represented by the magnitude of the database, extracted from the national Swedish Registry of Gallstone Surgery and ERCP (GallRiks), which describes the outcome of cholecystectomies performed at nearly all hospitals in Sweden in routine clinical practice. Thus, regional differences, as well as differences in catchment areas and profiles of the hospitals, can be compensated for. An additional strength of the study is that the data are registered prospectively by the respective surgeons, online, at the time of completion of the operation.

We also recognize that there are some obvious limitations of the study. GallRiks is a national quality register for gallstone surgery and ERCP, and accordingly, the quality and relevance of some clinical data can rightfully be questioned since these are not entered into the registry as a part of a randomized controlled trial protocol with the specific, dedicated aim of addressing specific questions. A further limitation of the register relates to the definition of the variable postoperative adverse event. Although AEs are defined in the Web protocol, these can be interpreted differently by the local coordinators at the participating hospitals. In order to minimize this risk, a list of the most common postoperative adverse events like pancreatitis, cholangitis, bile leakage, and deep venous thrombosis, just to mention a few, will from August 2016 and onwards be visible as predefined options to the local coordinator as an aid to minimize the interobserver variability. However, for the data presented in this study, the coordinator had to decide first whether a postoperative adverse event prevailed before the respective alternatives were chosen. Another possible limitation of these data is a possible risk of selection bias where the reporting surgeon may avoid reporting on negative adverse events related to the surgical intervention. To avoid this, special attention has been directed towards corresponding issues, in order to minimize loss of data quality. An independent coordinator at each participating hospital reviews each individual intervention and reports on imbalances at a compulsory 30-day follow-up. The GallRiks board also undertakes regular validation visits to all the participating hospitals, in order to ensure the accuracy and validity of the registered data. In order to adjust for the possible skewness of data and other confounding factors such as gender, age, comorbidity (American Society of Anesthesiologists classification scale), emergent or scheduled surgery, indication for and type of surgery, as well as a history of previous acute cholecystitis, these were managed through the multivariate analysis. It needs to be pointed out that the time elapsed from admission to the operation was only possible to calculate according to the final date of surgery by the date of admission. Accordingly, the exact time in hours between operation and surgery could not be captured. Likewise, the exact time from onset of symptoms of acute cholecystitis to admission is not registered in GallRiks. Finally, it must be emphasized that GallRiks is designed with the Swedish Health Care System in mind and that the findings in this study must be interpreted cautiously from an international perspective. Accordingly some aspects on the current problem related to the management of AC can, due to methodological limitations, only be addressed by indirect measures.

## Conclusion

The optimal timing of cholecystectomy for patients with acute cholecystitis appears to be within the first 2 days of hospital admission. However, the somewhat higher frequency of adverse events after operations performed on the admission day emphasizes the importance of optimizing the patient before surgery as well as ensuring that the highest surgical resources are available at the time of operation.
